# *Culex torrentium* Mosquito Role as Major Enzootic Vector Defined by Rate of Sindbis Virus Infection, Sweden, 2009

**DOI:** 10.3201/eid2105.141577

**Published:** 2015-05

**Authors:** Jenny C. Hesson, Jenny Verner-Carlsson, Anders Larsson, Raija Ahmed, Åke Lundkvist, Jan O. Lundström

**Affiliations:** Uppsala University, Uppsala, Sweden (J.C. Hesson, J. Verner-Carlsson, A. Larsson, Å. Lundkvist, J.O. Lundström);; Public Health Agency of Sweden, Solna, Sweden (J. Verner-Carlsson, R. Ahmed, Å. Lundkvist);; Nedre Dalälvens Utvecklings AB, Gysinge, Sweden (J.O. Lundström)

**Keywords:** Culex, Sindbis virus, arthropod vector, Europe, arthritis, Alphavirus, enzootic, vector-borne infections, mosquitoes, mosquitoborne, Sweden, viruses

## Abstract

We isolated Sindbis virus (SINV) from the enzootic mosquito vectors *Culex torrentium*, *Cx. pipiens*, and *Culiseta morsitans* collected in an area of Sweden where SINV disease is endemic. The infection rate in *Cx. torrentium* mosquitoes was exceptionally high (36 infections/1,000 mosquitoes), defining *Cx. torrentium* as the main enzootic vector of SINV in Scandinavia.

In Sweden, Finland, Russia, and South Africa, Sindbis virus (SINV; family *Togaviridae*, genus *Alphavirus*) is an etiologic agent for outbreaks of rash and long-lasting polyarthritis (*1*). Ecologically, SINV is a zoonotic mosquitoborne virus that naturally circulates in bird populations but only incidentally infects humans (*1*). Previous detections and isolations of SINV from field-collected mosquitoes identified the ornithophilic mosquitoes *Culex pipiens*/*Cx*. *torrentium* and *Culiseta morsitans* as possible enzootic vectors of SINV and the generalist mosquitoes *Aedes cinereus* and *Ae. rossicus*, which feed on birds and humans, as potential bridge vectors for transmission of the virus from viremic birds to humans (*2*,*3*; J.C. Hesson, J.O. Lundström, unpub. data). However, female *Cx. torrentium* and *Cx. pipiens* mosquitoes are morphologically indistinguishable, so all previous virus isolates from these species were from pools that may have contained both species. The distinction between *Cx. torrentium* and *Cx. pipiens* is necessary because vector competence experiments show great differences between the capacities of the 2 species to become infected with and to transmit SINV (*4*,*5*). *Cx. torrentium* is highly superior to *Cx. pipiens* as a vector of SINV in the laboratory (*4*,*5*), but the extent to which the 2 species are infected in nature is unclear.

We determined the natural SINV infection rates (IRs) in *Culex* mosquitoes, which were identified by using a newly developed molecular method for reliable identification of *Cx. torrentium* and *Cx. pipiens* mosquitoes (*6*). We also studied the simultaneous occurrence of SINV in *Cs. morsitans* mosquitoes.

## The Study

Every 2 weeks during July 13–September 13, 2009, we collected adult female mosquitoes by using 35 miniature light traps from the US Centers for Disease Control and Prevention (Atlanta, GA, USA) baited with carbon dioxide; the traps were set within the regular mosquito surveillance area of the River Dalälven floodplains in central Sweden (*7*). Mosquitoes were kept cold on a chilled table during morphologic identification and stored at −80°C. Legs were removed from mosquitoes morphologically identified as *Cx. pipiens*/*Cx. torrentium* and used for DNA extraction, enabling identification of the individual specimens to species by using a previously described molecular method (*6*).

RNA was extracted from 668 mosquito bodies without legs (301 *Cx. torrentium*, 367 *Cx. pipiens*) and from 74 pools of mosquitoes pooled by collection trap and week (290 *Cs. morsitans*); pool sizes ranged from 1 to 19 mosquitoes. The mosquitoes were processed for RNA extraction, real-time reverse transcription PCR (rRT-PCR), and virus isolation on Vero cells as previously described (*3*).

First, all samples were screened by SINV rRT-PCR by pooling 2–10 RNA extractions by species and collection week. Of 81 total pools, 14 were positive for SINV RNA. Nine positive pools were from *Cx. torrentium* mosquitoes, and 3 and 2 pools, respectively, were from *Cx. pipiens* and *Cs. morsitans* mosquitoes. Second, all individual samples from the SINV-positive pools from the first screening were subjected to another rRT-PCR, so that individual (*Culex*) or smaller pools of (*Culiseta*) mosquitoes were ultimately tested for SINV RNA. The second rRT-PCR showed 16 samples positive for SINV RNA. One of the positive *Cx. torrentium* pools, which contained samples from 10 mosquitoes, included samples from 3 SINV RNA–positive mosquitoes. Thus, 11 of 301 individual *Cx. torrentium* and 3 of 367 individual *Cx. pipiens* mosquitoes were positive for SINV RNA. For *Cs. morsitans* mosquitoes, 2 of 74 pools were positive for SINV RNA (Table 1).

**Table 1:** Ornithophilic *Culex* and *Culiseta* spp. mosquitoes collected for the detection of Sindbis virus, central Sweden, 2009

Mosquito species	No. collected (no. from which Sindbis virus isolated)							Total		Infection estimates
	Jul 14		Jul 28 and 29	Aug 11	Aug 27		Sep 8			
*Culex* spp.										
* Cx. torrentium*	45	183 (6)		62 (4)		9 (1)	2	301(11)	36.5†	
* Cx. pipiens*	38	134 (2)		175 (1)		13	7	367 (3)	8.2†	
Total	83	317 (8)		237 (5)		22 (1)	9	668(14)	21.0†	
*Culiseta* sp.										
* Cs. morsitans*	8	35		93		111 (1)	43 (1)	290 (2)	6.9‡	
Total	91	352 (8)		330 (5)		133 (2)	52 (1)	958 (16)	NA	

*NA, not applicable. †Infection rate/1,000 mosquitoes. ‡Minimum field infection rate/1,000 mosquitoes.

Because individual *Cx. torrentium* and *Cx. pipiens* mosquitoes were tested, the IRs were calculated as (no. positive individual mosquitoes/total no. tested) × 1,000; differences between the species were tested for significance by using a χ^2^ test. The actual species-specific IR differed significantly between species (p = 0.01): 36.5, 8.2, and 21 infections/1,000 mosquitoes for *Cx. torrentium*, *Cx. pipiens*, and mixed species (*Cx. pipiens and Cx. torrentium*), respectively. *Cs. morsitans* mosquitoes were tested in pools, so we calculated the minimum IR (MIR) as (no. positive pools/total no. mosquitoes tested) × 1,000; the calculated MIR was 6.9 infections/1,000 *Cs. morsitans* mosquitoes. A comparison of MIR and maximum-likelihood estimates of infection gave similar estimates.

SINV was successfully isolated from all 16 rRT-PCR–positive mosquito samples (Table 2). Part of the SINV E2 envelope glycoprotein gene was sequenced as previously described (*8*). The phylogenetic analysis included all genomic sequences obtained in this study together with previously published sequences for 63 other virus strains (*8*). The results showed that all new strains belonged to the SINV-I genotype and have close relationships with strains from Europe, the Middle East, and South Africa (Figure).

**Table 2:** Summary of 16 Sindbis virus isolates from ornithophilic *Culex* and *Culiseta* species mosquitoes collected in central Sweden, 2009

Species	Date collected	Geographic coordinates	No. mosquitoes	Strain	GenBank accession no.
*Cx. torrentium*	Jul 28	60°10.141′N; 16°34.998′E	1	09-M-526-3	KF297644
*Cx. torrentium*	Jul 28	60°10.141′N; 16°34.998′E	1	09-M-1393-3	KF297639
*Cx. torrentium*	Jul 28	60°14.698′N; 16°43.592′E	1	09-M-1388-17	KF297643
*Cx. torrentium*	Jul 28	60°7.506′N; 16°46.828′E	1	09-M-1384	KF297637
*Cx. torrentium*	Jul 28	60°7.506′N; 16°46.828′E	1	09-M-1394	KF297640
*Cx. torrentium*	Jul 28	60°6.253′N; 16°45.091′E	1	09-M-1396-1	KF297641
*Cx. torrentium*	Aug 11	60°10.141′N; 16°34.998′E	1	09-M-1367-3	KF297638
*Cx. torrentium*	Aug 11	60°9.647′N; 16°30.977′E	1	09-M-564-9	KF297646
*Cx. torrentium*	Aug 11	60°9.647′N; 16°30.977′E	1	09-M-571-40	KF297636
*Cx. torrentium*	Aug 11	60°9.638′N; 16°54.428′E	1	09-M-991-1	KF297653
*Cx. torrentium*	Aug 27	60°26.076′N; 17°22.583′E	1	09-M-358-5	KF297651
*Cx. pipiens*	Jul 28	60°3.127′N; 16°43.320′E	1	09-M-519-25	KF297652
*Cx. pipiens*	Jul 29	60°17.846′N; 16°50.486′E	1	09-M-648-2	KF297647
*Cx. pipiens*	Aug 11	60°9.647′N; 16°30.977′E	1	09-M-564-5	KF297645
*Cs. morsitans*	Aug 25	60°10.141′N; 16°34.998′E	4	09-M-1169	KF297642
*Cs. morsitans*	Sep 8	60°7.506′N; 16°46.828′E	3	09-M-887	KF297648

**Figure:**
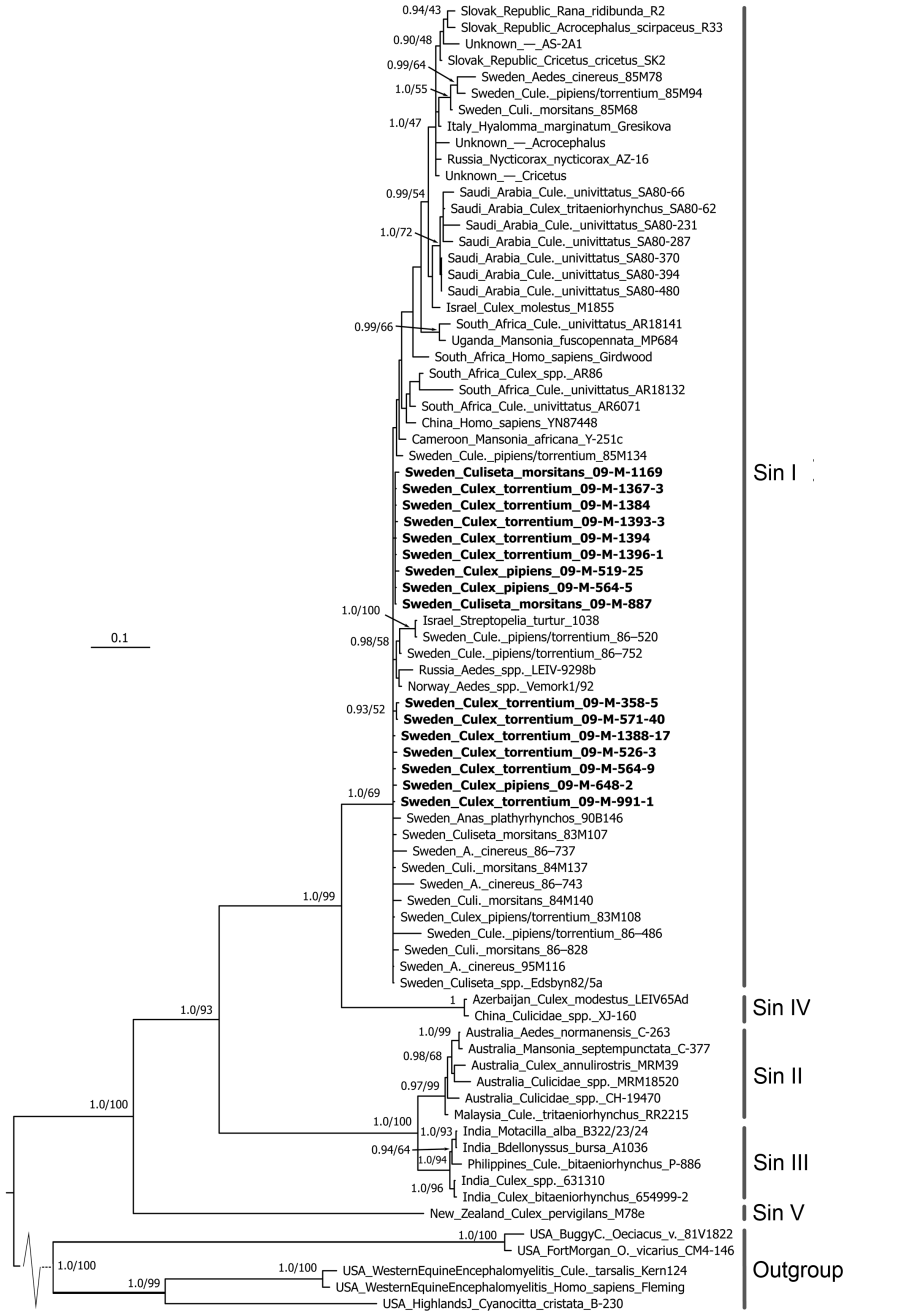
Consensus tree of the partial E2 envelope glycoprotein gene for Sindbis virus constructed by using MrBayes (http://mrbayes.sourceforge.net/). The phylogram includes 16 Sindbis virus strains isolated from mosquitoes collected in central Sweden during July 13–September 13, 2009, against a background of all Sindbis virus strains previously sequenced in the same region. The tree shows that all new strains are of the Sindbis-I virus genotype. Boldface indicates strains isolated during this study. Labels on right indicate Sindbis virus genotypes. Support values at nodes are Bayesian posterior probabilities/Garli maximum-likelihood bootstrap support. Nodes without support values have Bayesian posterior probability of <0.9, and branches are collapsed at 0.5 posterior probability. Scale bar represents average number of substitutions per site.

## Conclusions

We describe information on the actual occurrence and IR of SINV-I in the enzootic mosquito vectors *Cx. torrentium* and *Cx. pipiens*, reliably identified to species level. The significantly higher SINV-I IR observed for field-caught *Cx. torrentium* than *Cx. pipiens* mosquitoes is a key addition to the previous findings of the extreme susceptibility of *Cx. torrentium* mosquitoes to SINV-I (*4*). Experimental transmission studies showed that all infected *Cx. torrentium* mosquitoes could transmit the virus upon refeeding on a susceptible animal (*4*). Thus, the observed natural IR of 36.5 infections/1,000 *Cx. torrentium* mosquitoes translates to 36.0 mosquitoes/1,000 being able to transmit SINV-I. In contrast, because only one third of infected *Cx. pipiens* mosquitoes can transmit SINV-I upon refeeding, the observed natural IR of 8.2 infections/1,000 *Cx. pipiens* mosquitoes translates to only 2.0 mosquitoes/1,000 being able to transmit the virus (*4*).

The observed SINV-I IRs are very high for both species, and the IR for *Cx. torrentium* is among the highest ever reported for mosquitoes. This could partly be attributed to the fact that single mosquitoes were analyzed, as compared with the more common technique of pooling. This higher IR would have remained undetected if only pooled mosquitoes were analyzed, even though our original pools consisted of only 10 individual mosquitoes. *Cx. pipiens* mosquitoes are generally considered a secondary enzootic vector of SINV because, as in Sweden, they are less frequently found infected in nature in South Africa, Israel, and Saudi Arabia, where *Cx. univittatus* mosquitoes are the main SINV vector (*9*–*11*).

Because of the exceptionally high SINV IR for field-caught *Cx. torrentium* mosquitoes in Sweden, the outstanding vector competence results for the species in the laboratory (*4*), and its status as the dominating *Culex* species in SINV-endemic areas of Europe (*12*), *Cx. torrentium* can now be identified as the main enzootic vector of SINV-I in Scandinavia. In areas of Sweden and Finland where clinical SINV-I infections are most prevalent, *Cx. torrentium* mosquitoes account for >90% of the *Cx. pipiens/Cx. torrentium* population; in central Europe, where the virus is more uncommon in mosquitoes and no human cases have been observed, both species are equally common (*12*–*14*). Thus, a large population of *Cx. torrentium* mosquitoes may be a prerequisite for the intense enzootic transmission of SINV-I that is needed to increase the risk for spillover infections in humans. Whether the *Cx. torrentium* mosquitoes are also to be considered a vector of other mosquitoborne bird viruses remains to be investigated. In continental Europe, West Nile virus and Usutu virus are emerging, and it is unknown if *Cx. torrentium* has a vector role for these viruses is unknown because of the lack of transmission experiments and isolation attempts from reliably identified female *Cx. pipiens/Cx. torrentium* mosquitoes. Knowledge from such experiments and isolation attempts would be especially valuable for northern and central Europe where *Cx. torrentium* is the dominating candidate enzootic vector species for bird-associated viruses (*12*).
